# Upstream—News in Genomics

**DOI:** 10.1002/cfg.209

**Published:** 2002-10

## Abstract

This report on the literature spans from May to July, highlighting breakthroughs on several important genomes, including mouse, zebrafish, *Fugu* and *Plasmodium*. Recent papers have reported on a mechanism for genome size reduction in *Arabidopsis*, comparisons and verifications of large-scale protein–protein interaction datasets, developments in RNA interference approaches for mammalian systems and a solidphase peptide tagging method for proteomics.


**Comparative and Functional Genomics**
*Comp Funct Genom* 2002; **3**: 398–404.
Published online in Wiley InterScience (www.interscience.wiley.com). **DOI:** 10.1002/cfg.209

**Feature**

### Upstream — news in genomics

### Abstract

**This report on the literature spans from May to July, highlighting breakthroughs on several important genomes, including mouse, zebrafish, *Fugu* and *Plasmodium*. Recent papers have reported on a mechanism for genome size reduction in *Arabidopsis*, comparisons and verifications of large-scale protein–protein interaction datasets, developments in RNA interference approaches for mammalian systems and a solid-phase peptide tagging method for proteomics. Copyright © 2002 John Wiley & Sons, Ltd.**

### Genome/large-scale sequencing

On 6 May the NIH- and Wellcome Trust-funded International Mouse Genome Sequencing Consortium (IMGSC) announced that they had produced a draft of the mouse genome (Sanger Institute IMGSC Press Release). This project has benefited from knowledge gained and technological improvements that occurred as a result of the Human Genome Sequencing Project, being completed much more swiftly, in around a year. Although Celera previously completed a draft of the mouse genome, that data is not freely available to the public. The IMGSC's data, however, has been submitted to the major public databases. The draft is incomplete; ~4% of the genome remains to be determined and the current draft assembly will need some refinement. Their current analysis of the sequence of the 20 chromosomes gives an estimated genome size of 2700 Mb, which would make it slightly smaller than the human genome. They predict ~30 000 mouse genes, which again is slightly less than the average prediction for the human gene count. Some differences are immediately obvious, e.g. as one would expect for an animal with a more acute sense of smell, the mouse has far more olfactory receptor genes than human. Using the mouse data to aid in the finishing of the human genome should speed up that process, allowing the team to complete the human genome in time for the 50th anniversary of Crick and Watson's discovery of the structure of DNA, in April next year. The mouse genome should then be completed sometime in 2005.

On 9 May, a consortium from the Wellcome Trust Sanger Institute, The John Innes Centre, Warwick University (UK) and the National Yang-Ming University (Taiwan) presented the complete genome sequence of *Streptomyces coelicolor* A3(2) (Bentley *et al.*, 2002). They predict that the ~8.65 Mb linear chromosome (the largest bacterial genome to be completely sequenced so far) contains 7825 genes. Over two-thirds of naturally derived antibiotics come from the complex secondary metabolic pathways of the streptomycetes; the sequence revealed that there are more than 20 clusters of genes relating to the production of known or predicted secondary metabolites. The density of genes is essentially constant across the chromosome, with a slight decrease towards the termini. However, when looking at the roles of the genes, it becomes clear that there is a central core (comprising about half of the chromosome), which contains the vast majority of essential genes, whereas the arms mainly hold 'contingency' genes. It is only the central core that shows regions of synteny with other bacterial genomes, such as *M. tuberculosis* and *Corynebacterium diphtheriae* (other actinomycetes), implying that the arm regions were acquired after these lineages diverged. Within the large gene set of *S. coelicolor* are expanded gene families, many of which are involved in regulation, transport and degradation of extracellular nutrients; this most likely reflects its adaptation to its complex soil environment.

On 29 May a Japanese team reported on the construction and analysis of a full-length-enriched cDNA library from erythrocytic stage *Plasmodium*

Copyright © 2002 John Wiley & Sons, Ltd.

*falciparum* (the human malaria parasite; Watanabe *et al*., 2002). The paper describes the novel genes identified using the library and concludes that the transcripts of *Plasmodium* genes have diverse start sites. Comparing the cDNA sequences obtained with the complete sequence of *P. falciparum* chromosome 2 revealed three novel genes that were not identified by the computational annotation. The analysis of *P. falciparum* transcriptional start sites showed that they are longer on average, and far more diverse, than those of human transcripts. The authors propose that these observations could reflect mechanism(s) of gene expression that are unique to the AT-rich genome of *Plasmodium*.

In July, a group from Cornell University published their analysis of a large tomato EST collection, and six selected BAC clones from different parts of the tomato genome, in *Plant Cell* (Van der Hoeven *et al*., 2002). A collection of 120 892 single-pass ESTs derived from 26 different tomato cDNA libraries were reduced to a set of 27 274 unique consensus sequences (unigenes). 70% of the unigenes were shown to have identifiable homologues in the *Arabidopsis* genome. Those genes with metabolic functions were most conserved between the two genomes, whereas genes encoding transcription factors were amongst the fastest evolving. Most of the 10 largest conserved multigene families had similar copy numbers in tomato and *Arabidopsis*, suggesting that the expansion of these families occurred before the divergence of these two species. An exception to this was the E8-like protein family (associated with fruit ripening), which has higher copy number in tomato. Combining the data from the EST database and the six sequenced BACs led to the prediction that the tomato genome encodes ~35 000 genes.

In the 4 July issue of *Nature*, researchers from the Institute of Molecular Biotechnology (Germany) presented the sequence and analysis of chromosome 2 of *Dictyostelium discoideum* (Glöckner *et al*., 2002). This 8 Mb chromosome is the largest of the six *Dictyostelium* chromosomes, representing roughly one-quarter of the genome. Although it has a high A + T content (~80%), chromosome 2 has 2799 predicted protein coding genes and 73 transfer RNA genes. This gives a gene density of ~1 gene/2.6 kb, which is similar to that of *Schizosaccharomyces pombe*; only *Saccharomyces cerevisiae* has a higher gene density, at 1 gene/2 kb. Extrapolating to the whole genome using this gene density gave an estimated 11 000 genes in the *D. discoideum* genome. A significant number of the genes showed higher similarity to vertebrate genes than to those of other fully sequenced eukaryotes. The authors note that the genome is more similar to metazoans than to plants or fungi, supporting the view that the evolutionary position of *D. discoideum* is located after the divergence of the plant kingdom, but before the branching of fungi, from metazoa, placing it close to the base of metazoan evolution.

On 12 July, the Wellcome Trust Sanger Institute and the Washington University Genome Sequencing Centre (GSC) announced the release of a whole-genome shotgun (WGS) assembly of the genome of the nematode worm *Caenorhabditis briggsae*. *C. briggsae* is morphologically almost indistinguishable from *C. elegans*, although their lineages are estimated to have diverged approximately 100 million years ago. The GSC had sequenced 13 Mb of bacterial clones of the *C. briggsae* genome when the two centres agreed to shift to WGS reads. They have since sequenced ~1 million reads each from plasmids and BAC and fosmid ends, giving greater than 10× coverage. The sequence data, and BLAST servers, are available from both centres (*C. briggsae* genome project webpage at Sanger; *C. briggsae* sequencing webpage at the GSC) and the sequence will also be available from GenBank, EMBL and WormBase.

On 24 July Ensembl announced the availability of the first assembly of the zebrafish genome, with 3× coverage (Ensembl zebrafish webpage). The collaborative effort between The Wellcome Trust Sanger Institute and the zebrafish community (*Danio rerio* sequencing project webpage), which started in February 2001, has now provided enough data from BAC and PAC clones to construct a whole genome assembly. The sequences cover 36 Mb, which is ~2% of the 1.7 Gb zebrafish genome. EST assemblies from the Johnson Group at Washington University were used for BLAST searching and STSs from ZFIN and the Geisler laboratory in Tübingen have been mapped by e-PCR.

On 25 July, in a *Science* e-publication, an International Consortium reported on a WGS assembly and analysis of the 365 Mb *Fugu rubripes* genome (Aparicio *et al*., 2002). The assembly provides 95% coverage, with more than 80% of the sequence in multigene-sized scaffolds. Repetitive

Copyright © 2002 John Wiley & Sons, Ltd. 

DNA accounts for less than one-sixth, and gene loci take up about one-third, of this highly compact vertebrate genome. The gene distribution shows sparse and dense regions, as seen in the human genome. In contrast to the overriding observation of compaction of *Fugu* genes compared to human genes, some 'giant' genes are spread over significantly larger genomic regions than those of their human orthologues, despite having average coding sequence sizes. Approximately 75% of predicted human proteins were shown to have a strong match to *Fugu* genes, whilst the remainder had diverged significantly from pufferfish homologues or had none. The conserved linkages observed between *Fugu* and human genes indicate the preservation of chromosomal segments from a common vertebrate ancestor, but considerable scrambling of gene order has occurred.

### Comparative/evolutionary genomics

On 31 May a team from Celera (with the help of some academic researchers) published a draft sequence of mouse chromosome 16 and a comparison of this draft to the human genome (Mural *et al*., 2002). The 5.3× coverage was generated from samples from four mouse strains (A/J, DBA/2J, 129X1/SvJ and 129S1/SvImJ). The 19 788 scaffolds have been ordered and mapped onto the mouse chromosomes using public genetic and radiation hybrid maps. The comparison to the human genome showed that mouse chromosome 16 has seven human homology segments spread across six human chromosomes. This segmentation is the result of genomic rearrangements, but within the segments the DNA sequence order is strikingly conserved. 98.1% of the 11 822 'syntenic anchors' (short stretches of sequence showing a unique significant sequence match between the two species) found on mouse chromosome 16 are in the same syntenic chromosomal position, relative to adjacent anchors, in both human and mouse. This conservation of the order of the syntenic anchors implies that the rodent genome reorganization was one event, likely to have taken place after the divergence from the ancestor of primates, but before the divergence of the mouse and rat lineages. Also of note is that only 34% of the syntenic anchors fall in coding exons, and 44% are intergenic, implying that there has been some selective force against change in these sequences. Of the 731 genes on mouse chromosome 16,222 do not have a human homologue in the expected syntenic position; 164 of these have a homologue that does not match the expected position, and 44 are paralogues derived from rodent or primate specific gene duplications, the remaining 14 have no human homologues.

In the 28 June issue of *Science*, a team from the University of Uppsala (Sweden) and the University of Arizona (USA), presented a comparison of two fully sequenced genomes of *Buchnera aphidicola*, the obligate endosymbionts of aphids (Tamas *et al*., 2002). Their study revealed the most extreme genome stability observed to date. No chromosome rearrangements or gene acquisitions have occurred in these genomes in the past 50–70 million years. However, substantial sequence evolution and the inactivation and loss of individual genes were observed. Compared to the genomes of the closest free-living relatives, *Escherichia coli* and *Salmonella* spp., these genomes are more than 2000-fold less labile in content and gene order. The genomic stasis of *B. aphidicola* could be attributed to the loss of phages, repeated sequences and recA, and implies that *B. aphidicola* is no longer a source of ecological innovation for its hosts.

In the July issue of *Genome Research*, researchers from the John Innes Centre (UK) and Purdue University (USA) reported on a mechanism for reduction of genome size in *Arabidopsis* (Devos *et al*., 2002). There is great variation in the genome sizes of angiosperms, but whilst polyploidization and retrotransposon amplification are known to be major causes of genome expansion, there has been no evidence for opposing mechanisms that would stop unlimited genome growth. The group chose to look for evidence of genomic DNA loss in *Arabidopsis thaliana*, an angiosperm with a well-characterized and notably small genome. Their data indicate that illegitimate recombination is the main mechanism of genome size decrease in *Arabidopsis*, removing at least five-fold more DNA than unequal homologous recombination. They also suggest that presence of highly degraded retro-elements contradicts the theory (from the dating of intact retro-elements) that retrotransposon amplification in *Arabidopsis* has been confined to the last 4 million years. They now feel that the absence of repeats older than this could simply reflect their gradual degradation over time.

Copyright © 2002 John Wiley & Sons, Ltd.

### Bioinformatics

In the 3 May issue of *Science*, two researchers from the Brookhaven National Laboratory (USA) presented a study of the topological properties of interaction and regulatory networks (Maslov and Sneppen, 2002). They quantified correlations between connectivities of interacting nodes and compared them to a null model of a network, in which all links are randomly rewired. They found that for both interaction and regulatory networks, links between highly connected proteins were systematically suppressed, whereas those between highly connected and poorly connected pairs of proteins were favoured. They point out that this effect would decrease the likelihood of crosstalk between different functional modules of the cell and increase the overall robustness of a network by localizing effects of deleterious perturbations.

In the May issue of *Molecular Cell*, a group from Utrecht University (The Netherlands) and the EBI (UK) published a study combining interaction data with microarray data to increase the confidence in potential interactions and allow annotation for previously uncharacterized genes (Kemmeren *et al*., 2002). Co-expression of genes, determined by microarray experiments, was used to increase the confidence in 973 of 5342 putative two-hybrid interactions or interactions detected by purifying complexes in *S. cerevisiae*. Integrating the data from these two approaches enabled the team to provide functional annotation for over 300 previously uncharacterized genes. Experiments testing a cross-section of the *in silico* predictions proved the robustness of these approaches and the value of integrating data from different approaches.

In the 15 May issue of *Nucleic Acids Research*, a team from the National Center for Biotechnology Information (USA) reported on a study to delineate gene neighbourhoods across prokaryotic genomes (Rogozin *et al*., 2002). These neighbourhoods are usually not observed in their entirety in any one genome, but they can be detected as overlapping, partially conserved gene arrays, when looking across a number of bacterial or archaeal genomes. Searching for orthologous genes classified in the database of clusters of orthologous groups of proteins (COGs) in 31 prokaryotic genomes resulted in the identification of 188 clusters of gene arrays, which included 1001 of 2890 COGs. Mapping the clusters back onto actual genomes produced extended neighbourhoods, which were seen to include additional genes adjacent to the clustered genes, which are transcribed in the same direction. This resulted in a total of 2387 COGs being included in neighbourhoods. The majority of the neighbourhoods are mainly made up of genes that share a functional theme, but they also include a few genes with no clear connection to the main theme. The authors propose that although some of these genes might have unsuspected roles, others are maintained within gene arrays because of the advantage of expression at a level that is typical of the given neighbourhood, and call this phenomenon 'genomic hitchhiking'. The largest neighbourhood had 79 genes (COGs) and consisted of overlapping, rearranged ribosomal protein superoperons. The team note that genomic hitchhiking is particularly typical of this neighbourhood and other neighbourhoods with translation machinery component genes. Several of their neighbourhoods have identified previously unseen connections between genes, allowing new functional predictions, and further analysis showed that gene neighbourhoods appear to evolve via complex rearrangement, with different combinations of genes within a neighbourhood fixed in different lineages.

In the 23 May issue of *Nature*, a German/UK/USA team published a comparison of several large-scale protein–protein interaction data sets in yeast (von Mering *et al*., 2002). Taking two large two-hybrid interaction studies, the two recent mass spectrometric analyses of complexes, two sets of mRNA co-expression data and genetic interaction data comprising ~80 000 interactions, they found only 2400 that were supported by more than one method. They point out that are three possible explanations for this: the approaches may not have achieved saturation; many of the methods could be producing a significant fraction of false-positives; and some methods may have a limited ability to detect certain types of interactions. They then assessed the accuracy and coverage of each method in reproducing 10 907 trusted interactions derived from protein complexes annotated manually in the MIPS and YPD databases. None of the methods covered more than 60% of the proteins in the yeast genome and they note that the datasets show biases towards highly abundant proteins (except for the genetic approaches), towards evolutionarily conserved proteins and towards proteins from certain subcellular localizations.

Copyright © 2002 John Wiley & Sons, Ltd. 

In the June issue of *Genome Research*, a group of Chinese and US researchers described a property of Gramineae genes, and perhaps all monocotyledon genes, which is not observed in eudicotyledon genes (Wong *et al*., 2002). They have observed gradients in GC content, codon usage, and amino acid usage along the direction of transcription, beginning at the junction of the 5′-UTR and the coding region. These gradients are large enough to cause problems for the annotation of the rice genome and to hinder the detection of protein homologies across the monocotyledon–eudicotyledon divide.

In the 20 June issue of *Nature*, an international team published a global analysis of *Caenorhabditis elegans* operons (Blumenthal *et al*., 2002). *C. elegans* and its relatives are unique among animals in having operons. Operons are transcribed as multigene (polycistronic) pre-messenger RNAs and processed to monocistronic mRNAs using the specialized SL2 small nuclear ribonucleoprotein particle for downstream mRNAs. Although the complete *C. elegans* genome sequence revealed many gene clusters, only 28 operons have been reported. To find out how many of the clusters represent operons, they probed full-genome microarrays for SL2-containing mRNAs. This identified ~1200 genes, >90% of which are downstream genes, falling in 790 distinct operons. They predict that the genome contains at least 1000 operons, 2–8 genes long, comprising ~15% of all *C. elegans* genes. They also point out that as co-transcription of genes is often taken as evidence of potentially related function, their operon list could reveal previously unknown functional relationships.

### Functional genomics

In the 19 April issue of *Science*, a team from The Netherlands Cancer Institute reported on a new vector system (pSUPER), which directs the synthesis of small interfering RNAs (siRNAs) in mammalian cells (Brummelkamp *et al*., 2002). The use of RNA interference in mammalian systems has been hampered by cytotoxic responses to the introduced dsRNA and the lack of ability to generate stable loss-of-function phenotypes efficiently. The team showed that siRNA expression mediated by their vector caused efficient and specific downregulation of gene expression. Furthermore, the stable expression of siRNAs using the vector mediated persistent suppression of gene expression. The vector uses the polymerase-III H1-RNA promoter, which produces a small RNA transcript lacking a polyadenosine tail. The gene-specific insert consists of a 19 bp stretch of the gene of interest, followed by a nine-nucleotide spacer and then the reverse complement of the 19 bp stretch (which forms a stem–loop structure in the transcript).

This was followed in the May issue of *Nature Biotechnology* by three technical reports detailing very similar work on RNA interference in mammalian cells using small RNAs. In the first, a Japanese team presented a vector-based siRNA expression system that produces U6 promoter-driven siRNAs with four uridine 3′ overhangs, which can mediate RNA interference (Miyagishi and Taira, 2002). In the second paper, a team from the City of Hope, Duarte (Canada) used a U6 promoter construct to express functional double-stranded siRNAs following transfection into human cells (Lee *et al*., 2002). Both of these approaches use constructs in which the sense and antisense strands of the siRNA are transcribed from separate U6 promoters. The third paper, from a team at The University of Michigan (USA), described a very similar approach to Brummelkamp *et al*., in which a U6 promoter-driven construct produces a 19 bp siRNA stem, with the two strands linked by a loop (Paul *et al*., 2002).

In the 17 May issue of *Science*, a team from the Center for Plant Cell Biology (USA) showed that RNA silencing (the mechanism exploited by the RNA interference approach) could be induced by an animal virus (Li *et al*., 2002). They showed that flock house virus (FHV) initiates, and is a target of, RNA silencing in *Drosophila* cells. They also showed that FHV infection relies on suppression of this RNA silencing by an FHV-encoded protein, called B2. This demonstrated that RNA silencing is indeed an adaptive antiviral defence mechanism in animal cells. They went on to show that B2 could inhibit RNA silencing in transgenic plants, providing the first experimental evidence for the conservation of the RNA silencing pathway in the plant and animal kingdoms.

On 18 June two researchers from the University of California, Berkeley (USA) reported that they had found evidence for large domains of similarly expressed genes in the *Drosophila* genome (Spellman and Rubin, 2002). They searched *Drosophila* gene-expression profiles for over 80 experimental

Copyright © 2002 John Wiley & Sons, Ltd.

conditions for groups of adjacent genes that showed similar expression profiles. They uncovered ∼200 groups of adjacent similarly expressed genes, having between 10 and 30 members, that account for over 20% of *Drosophila* genes. The average group size was ∼100 kb, with sizes of 20–200 kb. No correlation could be found between group locations and chromosomal structures, and the genes within groups showed no obvious functional relationships, so the authors were unable to provide an explanation for this intriguing phenomenon. However, they did point out that in future comparisons with the *D. pseudoobscura* genome, these regions should show more synteny than other regions if the groupings do provide some selective advantage.

On 15 July an American group published the results of a combined bioinformatic and experimental approach to find conserved essential genes in *Streptococcus pneumoniae* (Thanassi *et al*., 2002). Candidate conserved genes were identified by comparing bacterial genomes and these were then deleted in *S. pneumoniae*, using a targeted disruption system in which a chloramphenicol resistance gene was introduced. 113 of the 347 candidate reading frames were presumed to be essential, due to lack of recovery of antibiotic-resistant colonies. The same high-throughput methodology was also used to overexpress gene products to look for possible polarity effects for the essential genes.

On 25 July a group from Stanford University (USA) used a near-complete collection of barcoded *Saccharomyces cerevisiae* gene deletants, in combination with a microarray loaded with the barcodes, to assess the requirement for each gene under six well-studied conditions: high salt, sorbitol, galactose, pH 8, minimal medium and nystatin treatment (Giaever *et al*., 2002). They showed that previously known and new genes were necessary for optimal growth under these conditions. Existing mRNA expression profiling data for the same yeast background strain in four of the tested conditions allowed them to determine that less than 7% of genes that exhibited a significant increase in mRNA expression were also required for optimal growth in these four conditions.

### Proteomics

In the May issue of *Nature Biotechnology*, a team from the Institute for Systems Biology (USA) described a method for site-specific, stable isotopic labelling of cysteinyl peptides in complex peptide mixtures through a solid-phase capture and release process, and the downstream isolation of those labelled peptides (Zhou *et al*., 2002). Recovered peptides were identified and (relatively) quantified by microcapillary liquid chromatography and tandem mass spectrometry (LC–MS/MS). They demonstrated the method by detecting galactose-induced changes in protein abundance in the yeast *Saccharomyces cerevisiae*. A comparison with the isotope-coded affinity tag (ICAT) method showed that the solid-phase peptide tagging method was simpler, more efficient, and more sensitive.

### Transcriptomics

In the 3 May issue of *Science*, a team from Affymetrix presented oligonucleotide array expression profiles across human chromosomes 21 and 22 using cytosolic polyadenylated RNA obtained from 11 human cell lines (Kapranov *et al*., 2002). The arrays were loaded with oligonucleotide probes spaced on average every 35 bp along the two chromosomes. When they compared their data with the existing annotations of chromosomes 21 and 22, they saw that as much as an order of magnitude more of the genomic sequence was transcribed than is accounted for by the predicted and characterized exons.

### Pharmacogenetics

In the 21 June issue of *Science*, an international team published a study of the structure of haplotype blocks in the human genome (Gabriel *et al*., 2002). They characterized haplotype patterns across 51 autosomal regions (covering 13 Mb of the human genome) in samples from Africa, Europe and Asia. They saw that the human genome could be broken down into haplotype blocks — regions in which there is little evidence for historical recombination and within which only a few common haplotypes are observed. Their data indicate that half of the human genome exists in blocks of 22 kb or larger in African and African–American samples and in blocks of 44 kb or larger in European and Asian samples. Within each block, a very small number of common haplotypes (three to five) are found on almost all chromosomes in

Copyright © 2002 John Wiley & Sons, Ltd. 

each population. The boundaries of blocks were seen to be highly correlated across populations. They then demonstrated that haplotype frameworks could provide substantial statistical power in association studies of common genetic variation across each region. These results should provide a foundation for building a haplotype map of the human genome, which could facilitate genetic association studies of human disease.

### References

Aparicio S, Chapman J, Stupka E, *et al.* 2002. Whole-genome shotgun assembly and analysis of the genome of *Fugu rubripes*. *Science* (e-publication ahead of print).

Bentley SD, Chater KF, Cerdeño-Tárraga A-M, *et al.* 2002. Complete genome sequence of the model actinomycete *Streptomyces coelicolor* A3(2). *Nature* **417**: 141–147.

Blumenthal T, Evans D, Link CD, *et al.* 2002. A global analysis of *Caenorhabditis elegans* operons. *Nature* **417**(6891): 851–854.

Brummelkamp TR, Bernards R, Agami R. 2002. A system for stable expression of short interfering RNAs in mammalian cells. *Science* **296**(5567): 550–553.

*C. briggsae* genome project webpage at Sanger: **http://www.sanger.ac.uk/Projects/C_briggsae/**

*C. briggsae* sequencing webpage at the GSC: **http://genome.wustl.edu/projects/cbriggsae/**

*Danio rerio* sequencing project webpage: **http://www.sanger.ac.uk/Projects/D_rerio/**

Devos KM, Brown JK, Bennetzen JL. 2002. Genome size reduction through illegitimate recombination counteracts genome expansion in *Arabidopsis*. *Genome Res* **12**(7): 1075–1079.

Ensembl zebrafish webpage: **http://www.ensembl.org/Danio_rerio/**

Gabriel SB, Schaffner SF, Nguyen H, *et al.* 2002. The structure of haplotype blocks in the human genome. *Science* **296**(5576): 2225–2229.

Giaever G, Chu AM, Ni L, *et al.* 2002. Functional profiling of the *Saccharomyces cerevisiae* genome. *Nature* **418**(6896): 387–391.

Glöckner G, Eichinger L, Szafranski K, *et al.* 2002. Sequence and analysis of chromosome 2 of *Dictyostelium discoideum*. *Nature* **418**(6893): 79–85.

Kapranov P, Cawley SE, Drenkow J, *et al.* 2002. Large-scale transcriptional activity in chromosomes 21 and 22. *Science* **296**(5569): 916–919.

Kemmeren P, van Berkum NL, Vilo J, *et al.* 2002. Protein interaction verification and functional annotation by integrated analysis of genome-scale data. *Mol Cell* **9**(5): 1133–1143.

Lee NS, Dohjima T, Bauer G, *et al.* 2002. Expression of small interfering RNAs targeted against *HIV-1* rev transcripts in human cells. *Nature Biotechnol* **20**(5): 500–505.

Li H, Li WX, Ding SW. 2002. Induction and suppression of RNA silencing by an animal virus. *Science* **296**(5571): 1319–1321.

Maslov S, Sneppen K. 2002. Specificity and stability in topology of protein networks. *Science* **296**(5569): 910–913.

Miyagishi M, Taira K. 2002. U6 promoter-driven siRNAs with four uridine 3′ overhangs efficiently suppress targeted gene expression in mammalian cells. *Nature Biotechnol* **20**(5): 497–500.

Mural RJ, Adams MD, Myers EW, *et al.* 2002. A comparison of whole-genome shotgun-derived mouse chromosome 16 and the human genome. *Science* **296**(5573): 1661–1671.

Paul CP, Good PD, Winer I, Engelke DR. 2002. Effective expression of small interfering RNA in human cells. *Nature Biotechnol* **20**(5): 505–508.

Rogozin IB, Makarova KS, Murvai J, *et al.* 2002. Connected gene neighborhoods in prokaryotic genomes. *Nucleic Acids Res* **30**(10): 2212–2223.

Sanger Institute IMGSC Press Release: **http://www.sanger.ac.uk/Info/Press/020506.shtml**

Spellman PT, Rubin GM. 2002. Evidence for large domains of similarly expressed genes in the *Drosophila* genome. *J Biol* **1**((1)): 5.

Tamas I, Klasson L, Canbäck B, *et al.* 2002. 50 million years of genomic stasis in endosymbiotic bacteria. *Science* **296**(5577): 2376–2379.

Thanassi JA, Hartman-Neumann SL, Dougherty TJ, Dougherty BA, Pucci MJ. 2002. Identification of 113 conserved essential genes using a high-throughput gene disruption system in *Streptococcus pneumoniae*. *Nucleic Acids Res* **30**(14): 3152–3162.

Van der Hoeven R, Ronning C, Giovannoni J, Martin G, Tanksley S. 2002. Deductions about the number, organization, and evolution of genes in the tomato genome based on analysis of a large expressed sequence tag collection and selective genomic sequencing. *Plant Cell* **14**(7): 1441–1456.

von Mering C, Krause R, Snel B, *et al.* 2002. Comparative assessment of large-scale data sets of protein–protein interactions. *Nature* **417**(6887): 399–403.

Watanabe J, Sasaki M, Suzuki Y, Sugano S. 2002. Analysis of transcriptomes of human malaria parasite *Plasmodium falciparum* using full-length enriched library: identification of novel genes and diverse transcription start sites of messenger RNAs. *Gene* **291**(1–2): 105–113.

Wong GK, Wang J, Tao L, *et al.* 2002. Compositional gradients in Gramineae genes. *Genome Res* **12**(6): 851–856.

Zhou H, Ranish JA, Watts JD, Aebersold R. 2002. Quantitative proteome analysis by solid-phase isotope tagging and mass spectrometry. *Nature Biotechnol* **20**(5): 512–515.

Upstream is a compilation of brief reports on papers and press releases of interest to our readers. They represent a personal critical analysis of the original content. If you would like to recommend a paper or newsworthy item, please contact our Managing Editor.

Copyright © 2002 John Wiley & Sons, Ltd. 
